# Diabetic Macular Ischemia Diagnosis: Comparison between Optical Coherence Tomography Angiography and Fluorescein Angiography

**DOI:** 10.1155/2016/3989310

**Published:** 2016-11-07

**Authors:** Jose Mauricio Botto de Barros Garcia, Talita Toledo Lima, Ricardo Noguera Louzada, Alessandra Thome Rassi, David Leonardo Cruvinel Isaac, Marcos Avila

**Affiliations:** Federal University of Goias, Av. Primeira Avenida, S/N, Setor Leste Universitario, 74605-020 Goiania, GO, Brazil

## Abstract

*Purpose*. To compare fluorescein angiography (FA) and optical coherence tomography angiography (OCTA) images of foveal avascular zone (FAZ) in patients with diabetic retinopathy (DR) with and without diabetic macular ischemia (DMI).* Methods*. The Wilcoxon signed-rank test was used to compare area measurements and *p* values of <0.05 were considered statistically significant. FA and OCTA images were independently graded by 2 observers that reached agreement regarding quantitative DMI according established protocols. The ischemic area was divided into “large” macular ischemia (superior to 0.32 mm^2^) and “small” (inferior to 0.32 mm^2^) groups. Quantitative analyses of the FAZ were performed using custom software.* Results*. Thirty-four eyes from 34 diabetic patients were enrolled. Subjects with DMI presented a mean area on FA and OCTA of 0.68 ± 0.53 mm^2^ and 0.58 ± 0.35 mm^2^, respectively (*p* = 0.1374). Patients without DMI presented a mean area on FA and OCTA of 0.19 ± 0.67 mm^2^ and 0.20 ± 0.79 mm^2^, respectively (*p* = 0.9594). The ICC for the FAZ measurements between the 2 observers on FA and OCTA was 0.96 and 0.92, respectively.* Conclusion*. OCTA represents a novel technique for the diagnosis of DMI and it may become an alternative to FA for this purpose.

## 1. Introduction

Diabetic retinopathy (DR) is a common complication of diabetes mellitus (DM) and is a leading cause of blindness worldwide [1]. Diabetic macular ischemia (DMI) is an important category of diabetic retinopathy (DR) [2, 3]. During the imaging study of the normal macula, an important hallmark is the capillary-free region called foveal avascular zone (FAZ) [2]. It is recognized that the FAZ can enlarge and can become irregular in DR and seems to get larger as the stage of retinopathy advances [2, 3]. DMI is characterized by the occlusion and loss of the macular capillary network or capillary dropout [4, 5]. This condition results in upregulation of growth factors, which contributes to the development of diabetic macular edema (DME), the most frequent sight-threatening disorder in individuals with DR [1]. DMI is an irreversible category of diabetic maculopathy, and its presence limits the potential benefits of treatments for DR [3, 5, 6]. The Early Treatment of Diabetic Retinopathy Study (ETDRS) established DMI standards that were determined using fluorescein angiography (FA). According to the ETDRS report 11, clinically, there is a correlation between DMI and poor prognosis that varies according to the severity of the macular ischemia. The anatomy of the retina appears to be altered in DMI, with thinning of retinal nerve fibre layer (RNFL) and outer retina and thickening of outer choroid [7].

In retinal microcirculation, blood supply is divided mainly into superficial capillary plexus (SCP) and deep capillary plexus (DCP) [8]. Choroidal circulation appears to be the most important blood supply to the central macula, including photoreceptor inner segment (IS) band, which is most likely the most important consumer of oxygen [9]. It is likely that the DCP is responsible for up to 15% of the blood supply to the photoreceptors, especially during dark adaptation [8, 10, 11].

FA has been the gold standard imaging modality since it was introduced in 1961 [7, 12, 13] However, it requires venipuncture, and reports of anaphylaxis and death related to contrast injections, despite being rare, have been documented [12]. In addition, the technique is costly and time-consuming, requiring up to 10 minutes for framing acquisition [4, 8, 10, 11]. Spectral domain (SD) OCT has emerged as a potential alternative for detecting macular nonperfusion in diabetic patients, but results are contradictory [1, 2, 13]. Macular ischemia may disrupt the normal flow of nutrients to the outer retina, but photoreceptor status on SD-OCT remains controversial [14]. In diabetic patients, the disruption of photoreceptors in SD-OCT can indicate a manifestation of underlying DCP nonperfusion in patients with a relatively healthy macula [14–16].

OCT angiography (OCTA) has been used for 3D mapping at microcirculation level [9, 13–16]. It allows detection of retinal and choroidal structures via motion contrast imaging and high speed scanning, which detect blood flow by analysing signal decorrelations between scans [7, 11, 14, 15]. Both inner and outer retinal capillary plexuses are imaged in contradistinction in conventional angiography, which does not effectively image the outer plexus [16].

This study aimed to compare the use of fluorescein angiography and OCTA in the diagnosis and quantification of DMI when applying a split-spectrum amplitude-decorrelation angiography algorithm (SSADA) [17] to improve the detection of flow signals in angiography with the purpose of offering a suitable “sparing” contrast alternative using 3 mm × 3 mm OCTA for clinical investigations of diabetic patients [8, 15–20].

## 2. Materials and Methods

This was a retrospective cross-sectional comparative study analysis conducted at Federal University of Goias and approved by Ethics Committee of the same institution. Signed informed consent was obtained from each subject prior to enrolment. This study was conducted in compliance with the tenets of the Declaration of Helsinki.

### 2.1. Inclusion Criteria and Data Collection

Imaging data were collected from patients who underwent FA and OCTA on the same day in a tertiary referral retina center in the period between January 1, 2015, and July 30, 2015. Exclusion criteria included significant cataract (without surgical indications at the time of examination), previous retinal arterial or venous occlusion, inherited macular dystrophy, posterior segment inflammation, and macular degeneration or scarring of any cause. Subjects that presented motion artifacts during OCTA or poor signal strength were also not included in this study. All subjects underwent a comprehensive ophthalmologic examination, including best-corrected visual acuity (BCVA) on ETDRS charts, +78 D noncontact lens slit-lamp fundoscopy, color fundus photography, and FA and OCT angiography, on a single day. Patients underwent OCTA with dilated pupils [18]. Color fundus photographs were obtained and FA was performed using a digital retinal camera (Topcon TRC _50_Dx; Topcon, Paramus, NJ). All patients received a standard infusion of 5 mL of 20% sodium fluorescein. Standard photographs were obtained at 20–40 seconds after contrast injection. The central macular nonperfusion area, FAZ boundaries, and the maximum height and area were manually delineated.

### 2.2. Acquisition and Analysis of Fluorescein Angiograms

The FAZ area was measured using a single FA photograph, such as the OCTA angiogram of the SCP and then divided into “large” (superior to 0.32 mm^2^) and “small” (inferior to 0.32 mm^2^) groups. SCP angiograms were selected because they provide the most reliable comparison with FA images. The “large” group comprised both “moderate” and “severe” ETDRS established grading groups [3, 19, 20]. The quantification of macular nonperfusion in both FA and OCT angiography captured images was completed using ImageJ software (ImageJ, National Institutes of Health, Bethesda, MD). No image manipulation was performed prior to FAZ area measurement.

### 2.3. Acquisition and Analysis of Optical Coherence Tomography Angiography

OCTA instrument used was the RTVue XR Avanti with AngioVue® software for OCTA (Optovue, Inc., Fremont, CA), and imaging data were obtained using the split-spectrum amplitude-decorrelation angiography (SSADA) [17] software. The algorithm was employed to improve the signal-to-noise ratio [8, 15–21]. This instrument operates at ~840 nm wavelength, 70,000 A-scans per second, and a bandwidth of 50 nm. The tissue resolution is 5 *μ*m axially and there is a 15 *μ*m beam width. The OCTA performs two repeated B-scans from 304 sequentially uniformly spaced locations. Each B-scan consisted of 304 A-scans for a total of 2 × 304 × 304 A-scans per acquisition, with a total acquisition time of approximately 3 seconds. The scanning area was captured in 3 mm × 3 mm sections, and the acquired OCT volumes were centered on the fovea [8–11]. A 3 mm × 3 mm OCTA image was used primarily because this size is the one that provides images with higher resolution in the used OCTA device [10, 11]. The SSADA algorithm was used to generate a volumetric rendering of blood flow from the internal limiting membrane (ILM) to the choroid, and it allowed the direct visualization of normal and abnormal blood circulation [18–21]. For the OCTA angiograms, it was used the automatic segmentation of the retinal layers at the level of the SCP generated by the AngioVue software in an orthogonal view. In order to correct for automated segmentation error and projection artifacts, the segmentation slab was manually adjusted, using corresponding structural OCT B-scans as a guide for the placement of 2 segmentation lines: the inner located at 3 *μ*m beneath the internal limiting membrane and the outer boundary at 15 *μ*m beneath the inner plexiform layer. This semiautomatic method permitted the readers to select images that pictured the largest extent of SCP for subsequent quantitative analysis. The DCP image was segmented with an inner boundary 15 *μ*m beneath the inner plexiform layer and an outer boundary at 70 *μ*m beneath the inner plexiform layer. This section captured both layers of the outer capillary plexus, which sandwiched the inner nuclear layer.

### 2.4. Statistical Analysis

All statistical analyses were performed using Statistical Package for Social Science 22.0 (IBM SPSS; IBM, Armonk, NY). FA and OCTA images were independently reviewed by two independent masked readers retina specialists (JG and DI) who reached an agreement regarding the area (mm^2^) of macular nonperfusion that was obtained using both methods according to ETDRS report 11. Intraclass correlation coefficient (ICC) was used to estimate the agreement between individual measurements from both readers. Since the ICC was consistently >0.9 between the 2 readers, nonparametric Wilcoxon signed-rank test was used to compare area measurements performed by 1 reader on FA and OCTA 3 mm × 3 mm scans.* p* values of <0.05 were considered statistically significant.

## 3. Results

### 3.1. Demographic Data

Thirty-four eyes from 34 patients, including 20 (58.82%) females and 14 (41.18%) males, were enrolled and separated according macular status (Table 1). Twenty-four eyes from 24 patients were placed in the group of patients with DMI, including 15 (62.5%) females and 9 (37.5%) males. Ten eyes with DMI (41.66%) had PDR. The mean (±SD) age of the DMI population was 61.20 ± 6.95 years. The group without DMI comprised 10 patients, including 5 (50%) females and 5 (50%) males. One patient (10%) had PDR. The mean age of this group was 64.09 ± 4.14 years.

### 3.2. Quantitative Analysis

**Figure 1:**
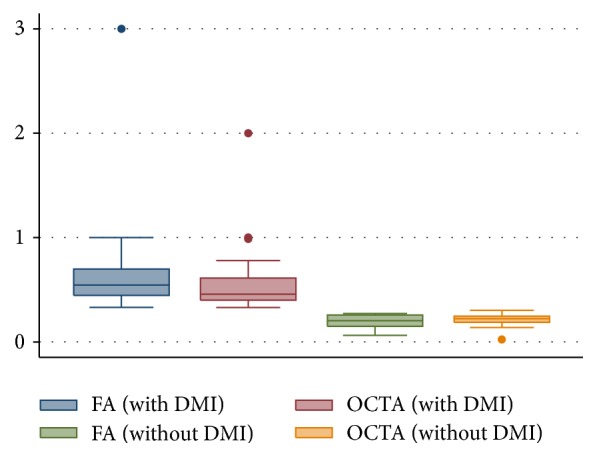
Box plots demonstrating foveal avascular zone (FAZ) area measurements on fluorescein angiography (FA) versus optical coherence tomography angiography (OCTA). Box blots demonstrate results according foveal avascular zone area on fluorescein angiography and optical coherence tomography angiography in patients with and without diabetic macular ischemia (DMI). Circles represent outliers.

Four eyes from 2 patients were excluded from the study due to a high quantity of motion artifacts. FAZ measurements were obtained for both groups (Table 2). Patients with DMI presented a mean FA of 0.68 ± 0.53 mm^2^ (95% IC, 0.46–0.90; *p* < 0.05). OCTA angiogram analysis demonstrated a mean of 0.58 ± 0.35 mm^2^ (95% IC, 0.43–0.73; *p* < 0.05). The Wilcoxon signed-rank test did not identify a significant difference between FA and OCTA in patients diagnosed with DMI (*p* = 0.1374). Patients without DMI presented a mean FA of 0.19 ± 0.67 mm^2^ (95% IC, 0.14–0.24; *p* < 0.05). OCTA angiogram analysis demonstrated a mean of 0.20 ± 0.79 mm^2^ (95% IC, 0.15–0.26; *p* < 0.05). The Wilcoxon signed-rank test did not identify a significant difference between FA and OCTA in patients without DMI (*p* = 0.9594) (Figure 1). The ICCs for FAZ area measurements between 2 observers with respect to FA and OCTA were 0.96 (IC: 0.36–0.71) and 0.92 (IC: 0.35–0.79), respectively, demonstrating the reproducibility and consistency of the methodology. This result highlights the notion that the FAZ is most often clearly demarcated by a distinct foveal vascular ring and that its abnormalities, in addition to its capillary dropout, can be lucidly obtained from OCTA images.

## 4. Discussion

Diabetic retinopathy is a microangiopathy that can develop DMI. ETDRS Research Group connected the severity of macular nonperfusion to the potential for progression in DR [2]. In fact, in DR, the advanced deterioration of macular perfusion is the basis for macular ischemia, and developing a method to perceive perfusion maps may allow correlations between central ischemia and the different stages of DR [17].

**Figure 2:**
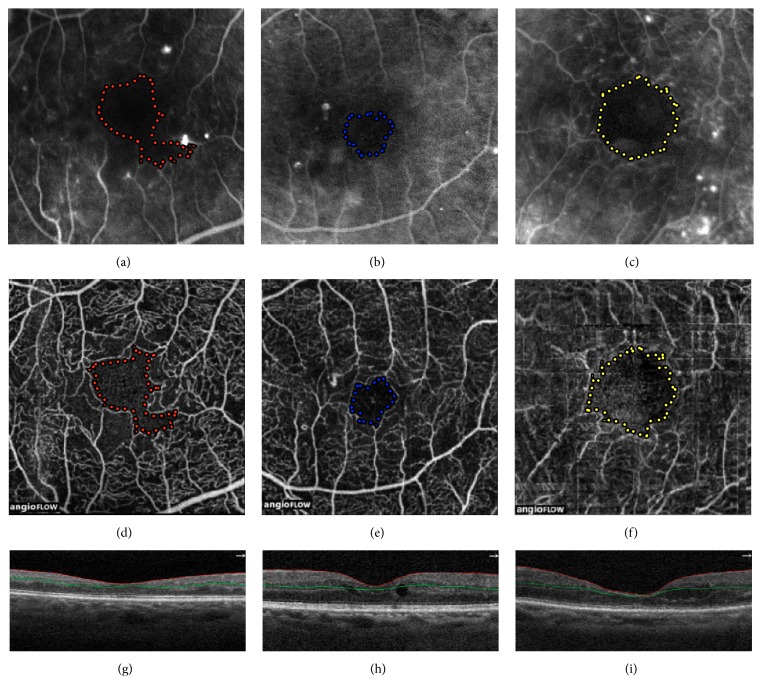
Diabetic macular ischemia (DMI) as seen on fluorescein angiography (FA) versus optical coherence tomography angiography (OCTA). Multimodal imaging of three different patients. Evaluation of foveal avascular zone (FAZ) extent on fluorescein angiography (a, b, and c) compared to optical coherence tomography (OCT) angiography. (d, e, and f) FAZ areas on both methods are represented by colored dotted lines. (g, h, and i) Semiautomatic segmentation of correspondent SD-OCT B-scan.

Fluorescein angiography is still considered the gold standard in retinal imaging on DR. However, it is an invasive method requiring venipuncture and contrast infusion; it is a time-consuming test and provides only 2-dimensional images. Therefore, reports of anaphylaxis and death related to contrast injections have been documented, despite being rare. The introduction of OCT modified the retinal disorders paradigm [13]. OCTA is a noninvasive method, obtains highly detailed 3-dimensional images without requiring injection of a contrast dye, and allows faster acquisition of images (Figure 2) [9, 10, 12–14].

**Figure 3:**
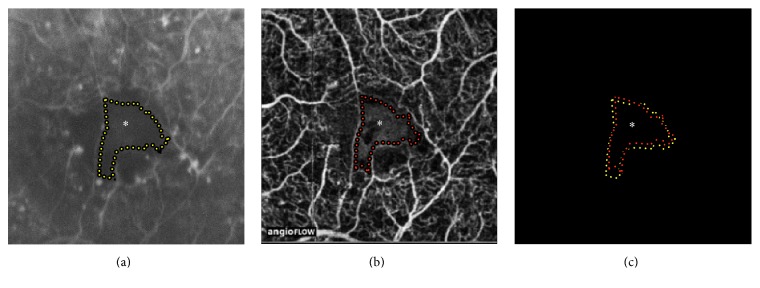
Quantifying foveal avascular zone (FAZ) area morphology on fluorescein angiography (FA) versus optical coherence tomography angiography (OCTA). Multimodal imaging of a left eye with central nonperfusion. (a) Yellow dotted lines delimits foveal avascular zone (FAZ) area on fluorescein angiography (FA) at 0:34 min. (b) Red dotted lines representing the FAZ area on optical coherence tomography angiography (OCTA) angiogram segmented at the level of the superficial retinal vasculature. (c) An overlap of FAZ areas obtained on FA and OCTA was performed, displaying similarity between the 2 measurements. To obtain centered images, a seed point at the center was used. To delimit FAZ areas in this specific case, the edge points were manually selected along the borders of the vessels, ignoring nearby capillary dropout region.

OCTA performed using a split-spectrum amplitude-decorrelation angiography (SSADA) algorithm has already been shown to be useful for imaging microvascular changes in DR [8–11, 14, 20]. Cole et al. also observed macular nonperfusion in a diabetic patient in a 3 mm × 3 mm OCTA that was centered on the fovea by applying a similar technology [20]. The 3 mm × 3 mm OCTA central sections obtained using SD-OCT allowed us to obtain a higher resolution over a small area (Figure 3). This area was sufficient for detecting central DMI, but it was not large enough to identify peripheral retinal nonperfusion. High-resolution OCT imaging allows measuring thickness of segmented retinal layers in angiographically apparent ischemic DR [22]. Future OCTA devices improvements may provide clinicians the ability to obtain wider field images with better resolution.

In the present study, statistical analysis also did not indicate significant difference between area measurements obtained with FA and OCTA in patients diagnosed with DMI. The same has occurred among patient without DMI regarding the measurement of normal FAZ area. The ICC for the ZAF area between the 2 observers on FA and OCTA demonstrated the reproducibility and consistency of used methodology. These results highlight the notion that FAZ is most frequently demarcated by a distinct foveal vascular ring and its abnormalities, in addition to its capillary dropout, can be lucidly obtained from OCTA imaging device [23].

Our study has limitations. First, all of the results were obtained during a single appointment, and there was no follow-up. The small sample size is another limitation. Further studies should be performed to provide support for these particular results.

The present study demonstrates that fluorescein angiography and OCTA provide similar results when used to diagnose macular ischemia in diabetic patients. With further improvements, OCTA may eventually reduce the need for fluorescein angiography [15].

## 5. Conclusions

In summary, OCTA may provide images with higher details regarding macular status, becoming a novel imaging technique for the diagnosis of DMI, and may become an alternative to FA for this purpose. The results also offer improved quantification of FAZ area in diabetic patients without DMI when compared to diabetic subjects with established macular ischemia.

## Figures and Tables

**Table 1:** Patient demographics and clinical and OCT data.

	Age		Gender		NPDR	PDR	BCVA (logMAR)	SD-OCT CRT (*μ*m)	
	Mean	SD	M	F			Mean	Mean	SD
With DMI (*n* = 24)	61.20	6.95	37.5%	62.5%	58.3%	41.7%	0.45	228	49.90
Without DMI (*n* = 10)	64.90	4.14	50.0%	50.0%	90.0%	10.0%	0.44	321	107.8

OCT: optical coherence tomography; SD-OCT: spectral domain optical coherence tomography; CRT: central retinal thickness; NPDR: nonproliferative diabetic retinopathy; PDR: proliferative diabetic retinopathy; BCVA: best-corrected visual acuity; M: male; F: female; SD: standard deviation; DMI: diabetic macular ischemia.

**Table 2:** Diabetic nonperfusion status and foveal avascular zone area on FA and OCTA.

	OCTA (mm^2^)		FA (mm^2^)		*p* value
	FAZ area		FAZ area		
	Mean	SD	Mean	SD	
With DMI (*n* = 24)	0.5851	0.3539	0.6850	0.5318	0.1374
Without DMI (*n* = 10)	0.2069	0.7990	0.1973	0.6787	0.9594

OCTA: optical coherence tomography angiography; FA: fluorescein angiography; FAZ: foveal avascular area; SD: standard deviation; DMI: diabetic macular ischemia. *p* value: calculated using nonparametric Wilcoxon signed-rank test.
